# Regulation of the Cell Cycle by ncRNAs Affects the Efficiency of CDK4/6 Inhibition

**DOI:** 10.3390/ijms24108939

**Published:** 2023-05-18

**Authors:** Qingyi Hu, Tao Huang

**Affiliations:** Department of Breast and Thyroid Surgery, Union Hospital, Tongji Medical College, Huazhong University of Science and Technology, Wuhan 430030, China; huqingyi@hust.edu.cn

**Keywords:** cell cycle, CDK, CDK4/6 inhibition, non-coding RNA, chemotherapy

## Abstract

Cyclin-dependent kinases (CDKs) regulate cell division at multiple levels. Aberrant proliferation induced by abnormal cell cycle is a hallmark of cancer. Over the past few decades, several drugs that inhibit CDK activity have been created to stop the development of cancer cells. The third generation of selective CDK4/6 inhibition has proceeded into clinical trials for a range of cancers and is quickly becoming the backbone of contemporary cancer therapy. Non-coding RNAs, or ncRNAs, do not encode proteins. Many studies have demonstrated the involvement of ncRNAs in the regulation of the cell cycle and their abnormal expression in cancer. By interacting with important cell cycle regulators, preclinical studies have demonstrated that ncRNAs may decrease or increase the treatment outcome of CDK4/6 inhibition. As a result, cell cycle-associated ncRNAs may act as predictors of CDK4/6 inhibition efficacy and perhaps present novel candidates for tumor therapy and diagnosis.

## 1. Introduction

In human cancers, abnormal cell cycles are often identified. The beginning and progression of the cell cycle are controlled by cyclin-dependent kinases (CDKs), cyclin, and cyclin-dependent kinase inhibitors (CKIs). The activated CDK4/6-cyclin D’s complex phosphorylates RB (pRB) upregulates the E2F’s transcription factor and promotes the expression of cell division-related target genes [1]. The entire cell cycle is monitored by checkpoints [2,3]. Checkpoints halt or delay the cell cycle when there is DNA damage, replication defects, or aberrant mitosis, ensuring the structural integrity of chromosomes. The G1/S checkpoint, which is the primary regulator of the cell cycle and essential for the onset and advancement of malignant tumors, is regulated by CDK4/6 [4]. Theoretically, it would be predicted that many human cancers will show increased CDK4/6 reliance and make ideal candidates for CDK4/6 inhibitor treatment since they often activate CDK4/6 genes or have transcriptional aberrations through a variety of mechanisms [5,6].

In some solid tumors, pharmacological inhibition of CDK4/6 has demonstrated substantial effectiveness, mostly by preventing Rb phosphorylation and causing G1 cell cycle arrest in tumor cells [7]. From laboratory investigations to clinical trials, these drugs have advanced swiftly. The Food and Drug Administration (FDA) has approved three medications (palbociclib (PD0332991) [8], ribociblib (LEE011) [9], and abemaciclib (LY2835219) [10]) for the treatment of metastatic breast cancer that specifically target CDK4/6. Although CDK4/6 inhibitions are still in the preclinical and early clinical stages, an increasing number of mechanisms of resistance to CDK4/6 inhibitions have been reported.

Non-coding RNAs (ncRNAs) are RNA molecules that do not have the function of coding proteins [11]. The human genome contains 93% DNA that may be transcribed into RNA, of which only 2% encodes proteins as mRNA and the remaining 98% are known as ncRNAs [12]. Non-coding RNA can be divided into different categories roughly based on their size [13]. Small ncRNAs important in cancer include microRNAs (miRNAs), transfer RNAs (tRNAs), and PIWI-interacting RNAs (piRNAs). At the other end of the size spectrum are long non-coding RNAs (lncRNAs), which are characterized by untranslated RNAs more than 200 nucleotides in length and include subclasses such as pseudogenes and circRNAs. MicroRNAs are short ncRNAs about 22 nucleotides long; the expression of other RNAs can be regulated by binding the 5′ end of miRNAs to complementary sequences in target RNA. The roles of tsRNA and piRNA in somatic tissues and cancers, as well as the exact mechanisms of their own biogenesis, remain to be elucidated. These small ncRNAs that are abnormally expressed in cancer may also be useful biomarkers. Compared with small ncRNA, lncRNA exhibits a wide range of mechanism diversity and more functions. CircRNAs are single-stranded, covalently closed RNA molecules. Although circRNA has been widely found in cancer, most mechanisms of action and function have yet to be further investigated. While ncRNAs lack the ability to encode proteins, they can influence the expression of other genes through a variety of mechanisms. NcRNAs regulate a variety of biological activities, including cell differentiation, proliferation, apoptosis, and migration [14]. During the development and progress of cancer, ncRNAs’ expression is frequently dysregulated [15,16]. In actuality, ncRNAs also regulate the abundance of several cell cycle regulators, which affect the cell cycle and proliferation. Moreover, cell cycle-associated mechanisms also control the production of ncRNAs.

Because ncRNAs are significant participants in the cell cycle, we hypothesize that they may also play a role in the clinical response to CDK4/6 inhibition. In this article, we systematically analyze how the two most significant ncRNA types, lncRNA and miRNA, regulate the cell cycle and their effects on CDK4/6 inhibition.

## 2. CDKs and Cell Cycle

**Figure 1:**
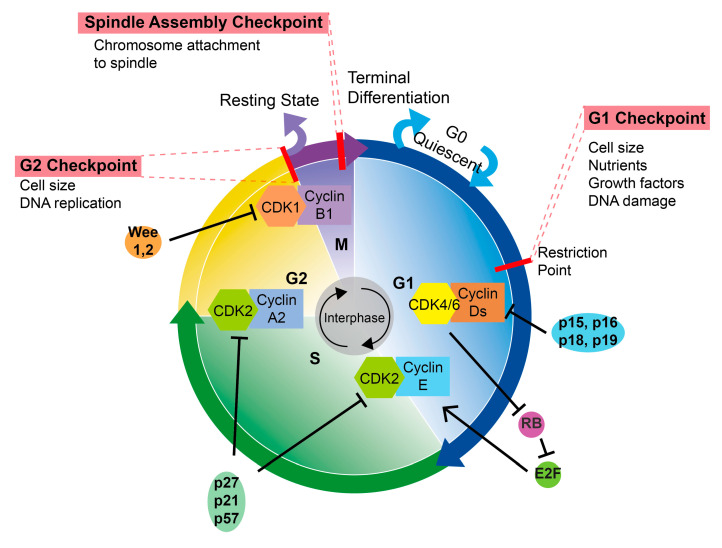
Outline of cell cycle control. The cell cycle is positively controlled by cyclin-dependent kinases (CDKs) and cyclins’ interactions and negatively controlled by cyclin-dependent protein kinase inhibitors (CKIs). S, DNA synthesis; M, mitosis; G1 and G2, index transition phase; G0, index quiescent status. Black lines end with arrow meaning promotion, black lines end with bar meaning inhibition, colorful arrows meaning cell cycle progression.

There are four stages in the cell cycle [17,18,19]: G1, S (DNA synthesis), G2, and M (mitosis). CDKs are an enzyme family that catalyzes protein phosphorylation during cell cycle progression and transcriptional control. CDK catalytic activity is dependent on cyclin interaction, and only particular CDK-cyclin interactions are expected to regulate the cell cycle process. Each phase of the cell cycle must be initiated under the control of cyclin, CDKs, and related pathways (Figure 1). RB suppresses cell cycle progression and proliferation by binding to and inhibiting the activity of E2F transcription factors [20,21,22]. At particular cell cycle stages, CDKs bind to and activate particular cyclins, which leads to the phosphorylation of target proteins and maintains cell cycle progression. It is possible to divide CDKs into two groups [23,24,25]: transcription-related CDKs (CDK7, CDK8, CDK9, CDK12, and CDK13), which directly control cell cycle progression, and cell cycle-related CDKs (CDK1, CDK2, CDK4, and CDK6), which are involved in the cell cycle.

CDK4/6 is thought to be essential for initiating the G1/S phase of the cell cycle. Growth factors, for example, often stimulate the MAPK pathway in the G1 phase and subsequently transcript genes encoding cyclin Ds, which bind and activate CDK4 and CDK6 [26]. The kinase activity of CDK4/6-cyclin Ds phosphorylates RB, and phosphorylation causes RB to separate from E2Fs [21,23]. To start transcription programs, E2F activates genes needed for the S phase and G2/M phase processes. At the late G1 phase, cyclin D1-CDK4/6 activity is reduced, whereas cyclin E-CDK2 activity rises. Cyclin E-CDK2 promotes the G1/S transition by phosphorylating RB and proteins involved in DNA replication, as well as many other important molecules involved in the initiation and progression of the S phase [27]. Cyclin E degrades during the early S phase, while the cyclin A-CDK2 complex accelerates the cell cycle process from the S to the G2 phase. CDK2 activity decreases throughout the middle G2 phase. The cyclin B/CDK1 complex phosphorylates APC/cyclosome and other target proteins to encourage G2 phase maturation and participate in M-phase activities [28]. After cytoplasmic division is completed in late M, cyclin B is destroyed, and the next cycle changes or enters a resting state. The progression of the cell cycle is monitored by numerous checkpoints, which interrupt or postpone the cell cycle in the event of DNA damage, replication faults, or aberrant mitosis.

The precise and timely regulation of CDK at each phase of the cell cycle also entails the negative regulation of several anti-proliferative signals, such as the expression of cyclin-dependent protein kinase inhibitors (CKIs). The N terminal of CKIs shares a common domain known as the kinase inhibition domain (KID) that works to inhibit CDKs. CKIs can specifically bind to distinct cyclin/CDK complexes, impede their activity, and impair the cell cycle. At present, there are two main CKIs, INK4 and CIP/Kip family [29,30,31].

The INK family comprises four proteins, p16^INK4a^ [32], p15^INK4b^ [33], p18^INK4c^ [34], and p19^INK4d^ [35], which compete with cyclin Ds to bind to CDK4/6, creating inactive heterodimers of the INK4 protein and CDK4/6 [36,37,38]. For instance, p16 and p15 contain ankyrin repeats to bind to CDK4 and CDK6, respectively. The INK family regulates the molecular basis of proliferation, differentiation, starvation, cell death, aging, and the development of acute and chronic diseases [39,40,41].

The Cip/Kip family contains p21^Cip1/Waf1^ (encoded by CDKN1A), p27^Kip^ (encoded by CDKN1B), and p57 ^Kip2^ (encoded by CDKN1C) [42,43,44,45]. The Cip/Kip protein, which is related or unrelated to CDK, plays a crucial role in gene transcription, apoptosis, autophagy, senescence, cytoskeletal remodeling, and cell mobility, and hence influences cell differentiation, development, organ biogenesis, and the occurrence and progression of cancer [46,47]. P21, for example, plays a significant role in the p53-dependent G1 block [42,48]. P21 binds to and inhibits cyclin/CDK activity, resulting in cell cycle growth arrest, and it also plays an environmentally dependent role in apoptosis [49,50].

In addition to INK4 and Cip/Kip, some kinases act as cell cycle checkpoints by negatively regulating CDKs. Wee1/2, also known as the Wee1A and Wee1B, work as DNA damage and cell volume checkpoints, and can inhibit CDK1 activity and cause G2/M arresting [51,52]. During DNA damage, cell cycle arrest induced by Wee1/2 is conducive to repairing damaged DNA and maintaining chromatin integrity.

## 3. LncRNAs and Cell Cycle

### 3.1. LncRNA Profile

Long non-coding RNAs (lncRNAs), which make up 80–90% of all ncRNAs, are defined as ncRNAs length 200 nucleotides—10 kb. The primary transcriptional byproducts of the human body are lncRNAs. The human genome’s intergenic regions include the majority of the DNA regions that code for lncRNAs. LncRNAs can also originate from sense or antisense transcripts with specific sequences that overlap protein-coding genes. LncRNAs are transcribed by RNA polymerase II and resemble mRNA structurally in many ways. The sequences of lncRNAs typically have a 3′-terminal polyA tail, a 5′-terminal 7-methyluridine (m7G) cap, and a promoter, but they lack an open reading frame (ORF) and thus have no protein-coding ability [53,54]. LncRNAs can be divided into five categories according to their transcription positions and directions: sense, antisense, bidirectional, intronic, and intergenic lncRNAs [55,56].

Although they do not directly encode proteins, lncRNAs play their biological functions by participating in epigenetics, transcription, post-transcriptional regulation, and other gene expression regulation at different levels, as well as various molecular mechanisms. LncRNA expression is abnormal in a variety of diseases [57,58,59]. For example, the lncRNA expression profiles of many tumor cells show significant changes compared with those of normal cells. Different lncRNAs have different functions; some are tumor suppressor genes, and some are tumor-promoting genes. LncRNAs are abnormally expressed in cancer, and the expression level can predict patients’ response to treatment and affect patients’ recurrence, metastasis, and prognosis [60,61,62].

LncRNAs influence downstream gene expression by cis and trans epigenetic mechanisms such as DNA methylation, chromatin remodeling, and histone modification. For example, the lncRNA XIST inactivates the X chromosome, which results in the cis-accumulation-mediated silencing regulation of a single X chromosome in XX female mammals [63]. LncRNAs can interact directly or indirectly with DNA and chromatin proteins to control chromatin structure. LncRNAs can function as ligands and bind to transcription factors to create complexes that regulate the activity of genes during transcription. Some lncRNAs are transcription factors [64,65]. LncRNA transcription will interfere with surrounding coding gene transcription, or lncRNA transcripts will influence the expression of neighboring coding genes. The DNA sequences of lncRNAs can interact directly with other DNA regions, changing their structure and potentially activating or inhibiting gene expression. LncRNAs can directly participate in the post-transcriptional control of mRNA via variable splicing, RNA editing, protein translation, and transport [60,66,67,68]. Antisense lncRNAs are primarily involved in the post-transcriptional control of coding genes [69,70]. Antisense lncRNAs can interact with mRNA complementary areas, influence the recruitment of splinters by some splicing sites, regulate the mRNA splicing process, and also influence the RNA editing process. LncRNAs can interact with proteins to help or inhibit the development of organelle structures, which can impact translation efficiency [71]. Furthermore, mRNA and lncRNA interactions can influence mRNA nuclear transport and intracellular distribution [72,73]. LncRNAs can also attach to miRNAs and stop them from binding to their target genes [74].

### 3.2. Cell Cycle Regulated by LncRNAs

**Figure 2:**
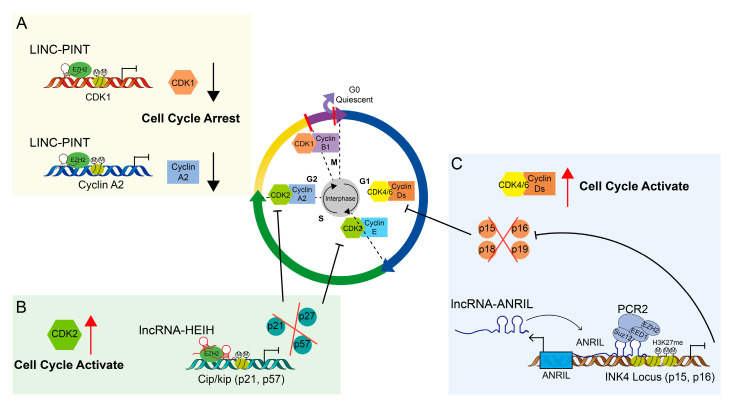
Schema showing the mechanisms of lncRNA-mediated regulation of the cell cycle via recruiting EZH2. (**A**). The model of LINC-PINT recruits EZH2, which induces H3K27me3, thus inactivating the transcription of CDK1, cyclin A2, AURKA, and PCNA genes. (**B**). A model of lncRNA-HEIH interacts with EZH2 to inhibit p15, p16, p21, and p57, thus leading to HCC tumorigenesis. (**C**). The model of lncRNA-ANRIL interacts with the PRC2 complex and recruits it to the INK4 locus to methylate histone H3K27. S, DNA synthesis; M, mitosis; G1 and G2, index transition phase; G0, index quiescent status. Red arrows upward meaning cell cycle activating, red arrows downward meaning cell cycle arresting; black lines end with arrow meaning promotion, black lines end with bar meaning inhibition.

LncRNAs can influence cell cycle progression via epigenetic regulation. LINC-PINT [75,76] recruits EZH2 to the promoters of target genes (CDK1, cyclin A2, AURKA, and PCNA), inducing trimethylation of H3K27 and epigenetic silencing of target genes, finally limiting the formation of melanoma (Figure 2A). Yang et al. [77] reported that lncRNA-HEIH is overexpressed in HBV-associated hepatocellular carcinoma. It interacts with EZH2 to negatively control the expression of CKIs p15, p16, p21, and p57. Hence, it plays an important role in the G0/G1 block (Figure 2B). According to reports, ANRIL [78] is linked to senescence and proliferation, as well as to the epigenetic regulation of INK4 transcription (Figure 2C). Yap et al. [79] showed that the PRC2 and ANRIL complex was involved in the regulation of cellular senescence by mediating the methylation of histone H3K27 at INK4 via EZH2. Kotake et al. [80] reported that ANRIL depletion causes human fibroblast growth arrest and increases age-related beta-galactosidase expression.

Cyclin and CDKs are key regulators of the cell cycle and are usually regulated by lncRNAs [81]. CDK1 expression can be positively regulated by lncRNA TMPO antisense transcripts (TMPO-AS) [82]. Downregulation of TMPO-AS1 reduced lung cancer cells’ viability, increased cyclin D1 and BCL expression, and triggered apoptosis. TMPO-AS1 is expected to provide a novel therapeutic target for lung cancer patients. A CDK1-related lncRNA is LINC00261 [83]. LINC00261, CDK1, and CXCL8 have been found in studies to have a mutual regulatory connection, impacting tumor angiogenesis, G2/M phase transition, and EMT. They promote tumor growth and impair immunotherapy efficacy. LINC00630 increases E2F1 binding to the CDK2 promoter region, stimulates CDK2 transcription, and thereby accelerates the malignant development of liver cancer [84]. LINC00630 is a promising molecular target for liver cancer treatment. ARAP1-AS1 stimulated cyclin D1 expression but did not affect CDK4 and CDK6 expression [85]. ARAP1-AS1 knockdown dramatically reduced lung cancer cell proliferation and clonogenicity and caused G0/G1 cell cycle arrest via reducing cyclin D1 expression.

Some lncRNAs have the potential to influence the expression of CDKs and cyclins at the same time. ALMS1-IT1 can activate the CDK pathway, increasing the proliferative potential of lung cancer cell lines and promoting malignant development [86]. LncRNA ENST00000512916 was shown to be upregulated in ameloblastoma (AB) tissues [87]. It elevates CDK2/4/6 expression in AB cells, enhancing cell cycle progression and proliferation.

CKIs’ expression is essential for controlling the cell cycle, and certain lncRNA can control both CKIs’ and CDKs’ expression simultaneously to improve cell cycle control. Maternally expressed gene 3 (Meg3) expression levels are significantly lower in non-small cell lung cancer (NSCLC) [88]. Meg3 facilitates miR-3163’s interaction with the 3′-UTR of the Skp2 mRNA, which prevents Skp2 from being translated. Skp2, a component of the E3 ubiquitin ligase SCF, particularly encourages p27’s destruction by ubiquitination. TPT1-AS1 (tumor protein translation control 1 antisense RNA1) has been linked to the development of cervical cancer, ovarian cancer, gastric cancer, and other cancers [89,90,91,92]. According to studies, TPT1-AS1 negatively regulates the expression of p21 while positively regulating the expression of CDK4 and cyclin D1. TPT1-AS1 is crucial for encouraging cell growth and controlling the G1/S transition in the cell cycle. It is possible to create more effective molecular therapy targets for TPT1-AS1. The lncRNA ABHD11-AS1 was found to be overexpressed in gastric cancer, ovarian cancer, bladder cancer, and endometrial cancer. According to research [93], the lncRNA ABHD11-AS1 upregulates Cyclin D1, CDK1, CDK2, CDK4, and Bcl-xl, downregulates p15, and increases cell proliferation and the G1/S transition while inhibiting cell death in endometrial cancer. The relationship between variations in lncRNA expression and the prevalence of triple-negative breast cancer has been discussed in previous studies. The lncRNA ZFAS1 was dramatically downregulated in breast cancer patients’ blood samples and reduced tumor cell proliferation and metastasis by positively influencing the expression levels of CDK inhibitors p21 and p27 [94]. CADM1-AS1 (cell adhesion molecule 1-antisense RNA 1, long non-coding RNA) suppresses AKT and GSK3b phosphorylation in HCC cells [95]. By inhibiting the AKT/GSK3b signaling pathway, CADM1-AS1 increases the expression of p15, p21, and p27 while decreasing the expression of cycle-related proteins such as cyclin Ds, cyclin Es, CDK2, CDK4, and CDK6, which prevents the transition from G0/G1 to S phase both in vitro and in vivo.

DNA damage is a crucial event that triggers checkpoints to stop or slow the cell cycle. The expression of several lncRNAs changes in response to DNA damage, controlling the expression of genes involved in the cell cycle. Gadd7 is a lncRNA that controls CDK6 expression post-transcriptionally [96]. Gadd7 controls CDK6 expression negatively and serves as a translational regulatory factor. Gadd7 participates in the G1 checkpoint and downregulates the cyclin D1/CDK6 complex in cisplatin-mediated DNA damage, preventing the cell cycle from responding to DNA damage [97]. More research is necessary because this might be a brand-new G1 checkpoint cascade.

**Figure 3:**
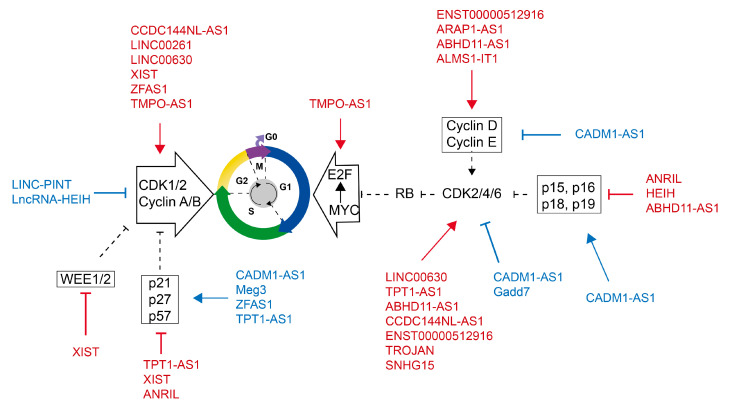
An overview of cell cycle control by lncRNAs. Lines exhibiting lncRNAs that accelerate proliferation are in red, whereas lines showing lncRNAs that reduce cell cycle are in blue. Lines ending with arrows indicate lncRNA promotion on specific molecules. Lines ending with a bar indicate lncRNA inhibition on downstream molecules. Dash lines indicate the regulation of CDKs, cyclins, and CKIs within the cell cycle. S, DNA synthesis; M, mitosis; G1 and G2, index transition phases; and G0, index quiescent status.

Some studies have discovered that while lncRNAs control the cell cycle, they also have impacts on the expression of particular miRNAs. XIST increased PC cell line proliferation by being positively correlated with CDK1 and negatively correlated with miR-140 and miR-124 [98]. XIST knockdown caused cell cycle stalling in the G1 phase, lowered p53 and CDK1 protein levels, and elevated p21 protein levels, limiting PC cell line proliferation. XIST can be exploited as a therapeutic target for human pancreatic cancer. In addition, lncRNA and miRNA can work together to control the expression of specific genes. The level of the lncRNA CCDC144NL-AS1 was markedly increased in liver cancer tissues [99]. Through the adsorption of miR-940 via sponging, CCDC144NL-AS1 stimulates the production of WDR5, and since CDK1, CKD2, and CDK4 are all downstream targets of WDR5, this promotes the development of liver cancer. In general, lncRNAs regulate cell cycle progression in a variety of ways (Figure 3 and Table 1).

## 4. MiRNAs and Cell Cycle

### 4.1. MiRNA Profile

MicroRNAs (miRNAs) are small, single-stranded ncRNAs that have a length of 20–24 nt. MiRNAs are abundant in eukaryotes and can make up between 1 and 4% of the genes in humans [102].

Among the small ncRNA species, miRNAs are by far the most extensively studied in cancer, compared to tsRNA and piRNA. MiRNA has been found to be altered in all cancer types studied, and the alteration of miRNA has important effects on the molecular and cellular processes of cancer [103,104,105].

MiRNAs are transcribed by RNA polymerase I and RNA polymerase II, with RNA polymerase II being the most common. Long, double-stranded primary miRNAs (pri-miRNAs) are the origins of miRNAs. Pri-miRNA is detected in the nucleus by DGCR8 (Drosha chaperone) and cleaved by RNase iii (Drosha) to form a miRNA precursor (pre-miRNA) [106]. The pre-miRNA is subsequently processed in the cytoplasm by the RNase iii endonuclease Dicer to generate mature miRNA molecules, which are generally 22 nucleotides long [107,108]. One strand of RNA is destroyed, and another is loaded into the AGO protein from the 5 or 3 ends, generating a miRNA-induced silencing complex (miRISC) [109]. The name of mature miRNA is 5p or 3p, depending on the directionality of the miRNA strand [110,111].

Through base-pairing with the 3′-UTR or CDS (coding DNA sequence) of mRNA, miRNA can silence a gene by causing the degradation of the target mRNA or preventing the target mRNA from being translated [112,113]. A single miRNA molecule can target many mRNAs, and multiple miRNAs can target the same mRNA, resulting in a complex network of miRNA–mRNA interactions [107,114]. Up to 30% of human protein-coding genes are under the control of miRNAs. Many biological processes, such as cell division, proliferation, apoptosis, and migration, are regulated by miRNAs. Moreover, they affect the body’s metabolism, growth, stem cell regeneration, and the emergence of cancer [115,116].

Compared to normal tissues, the expression of many miRNAs is dysregulated in tumors, indicating that miRNA plays a crucial role in the development and propagation of malignancies [105,117]. MiRNAs have been shown to have antitumor effects; for instance, miR-15 and miR-16 are downregulated in B-cell chronic lymphoma [118], miR-122 is downregulated in liver cancer cell lines [119,120], and miR-125 is downregulated in breast cancer [121]. MiRNAs can work as oncogenes in vivo. MiR-23a/24-2/27a clusters, on the other hand, enhance the invasion of breast cancer cells and liver metastases, whereas miR-10b promotes angiogenesis and metastasis in breast cancer [122,123]. Certain miRNA levels in the circulatory system can also reflect the malignancy of tumors, making them more useful detection markers for preoperative diagnosis and recurrence monitoring of patients [124,125]. MiRNAs can be employed as prognostic predictors in various malignancies. MiRNAs can be employed as therapeutic targets in the treatment of cancer.

### 4.2. Cell Cycle Regulated by MiRNAs

**Figure 4:**
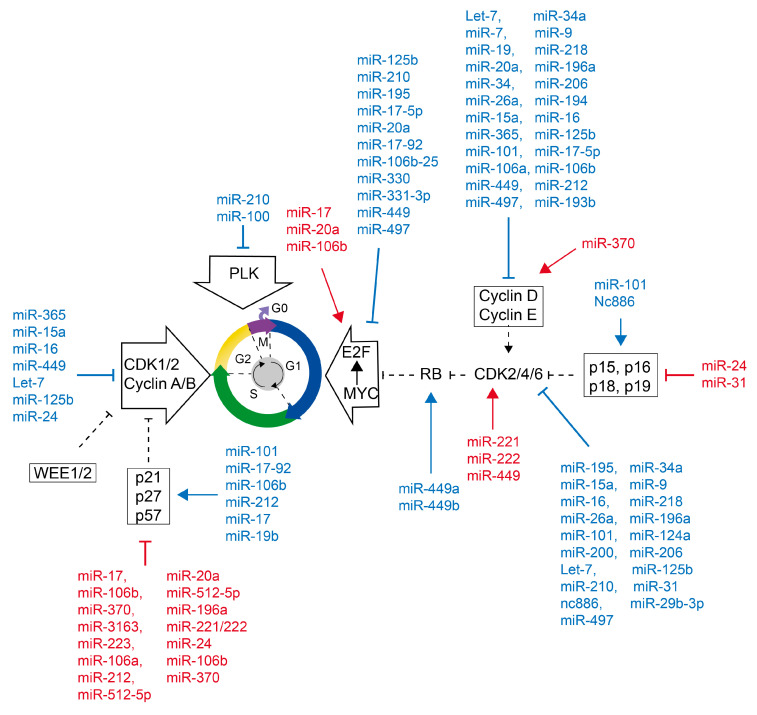
An overview of cell cycle control by microRNAs. Lines displaying miRNAs that accelerate proliferation are in red, whereas miRNAs that reduce cell cycle are in blue. Lines ending with an arrow indicate miRNA promotion on downstream molecules. Lines ending with a bar indicate miRNA inhibition on downstream molecules. Dash lines indicate the regulation of CDKs, cyclins, and CKIs within the cell cycle. S, DNA synthesis; M, mitosis; G1 and G2, index transition phase; G0, index quiescent status.

Some studies have revealed that miRNAs can influence the levels of a wide range of cell cycle regulators (Figure 4 and Table 2). Cell cycle regulators are precisely regulated by miRNAs at the translational and post-translational levels in addition to transcriptional levels, having an impact on a variety of signaling pathways and cellular activities. In mammalian cell cycles, miRNAs are expected to play a significant role and may even regulate cell division and proliferation.

MiRNAs may regulate G1/S transitions by targeting important cell cycle regulators such as CDK1, CDK2, CDK4, and CDK6, as well as cyclin D1, D2, and E. MiR-195 binds to the 3′-UTR of CDK6 mRNA to negatively regulate CDK6 expression in gastric cancer. MiR-195 targets and suppresses cyclin D3 in non-small cell lung cancer (NSCLC), resulting in cell cycle arrest in the G1 phase [126]. The miR-124a promoter is substantially methylated and downregulated in ALL, leading to an increase in CDK6 expression and the phosphorylation of RB [149]. Promoter methylation and p53 activity control the expression of miR-125a and miR-125b in liver cancer [152,153]. MiR-125a and miR-125b suppress cyclin D1 and cause p21-dependent cell cycle arrest in the G1 phase. In several cancers, miR-17-5p prevents cyclin D1 expression and causes G1-phase arrest [144,167,168,169]. MiR-449b inhibits the expression of cyclin D1 and E2F3 by binding to their 3′-UTRs, which reduces the growth of colon cancer stem cells and has tumor-suppressive effects [184]. CDK1 is a functional target of miR-31-5p that plays an important role in cell cycle control [185,186]. MiR-212 expression specifically targets cyclin D3 and promotes apoptosis in ATL cells, causing an increase in G0/G1 cells and a decrease in S-phase cells [215]. In melanoma cells, miR-206 causes G1 arrest by inhibiting the translation of CDK4, cyclin D1, and cyclin C [211,212]. MiR-365 acts as an intermediary, downregulating cyclin D1 and cdc25A via lowering p53 abundance [130,131,216]. MiR-15a [132] and miR-16 [217,218] are part of the mir-15a-16-1 cluster [133,134,135], which downregulates CDK1, CDK2, and CDK6 as well as cyclin D1, cyclin D3, and cyclin E1 to induce cycle stagnation in the G1 phase. MiR-26a significantly decreased the expression of EZH2 and repressed the expression of c-Myc, cyclin D3, cyclin E2, and CDK4/6 in an EZH2-dependent manner [156,157,158]. Furthermore, miR-26a can directly upregulate the expression of p14 and p21 and downregulate the expression of cyclin D2 without the need for EZH2. It is essential for halting the cell cycle’s development and reducing cell proliferation. MiR-7 [136,140,141], miR-19a [142,219], miR-20a [144,145,170], and miR-34 [147,220] all downregulate cyclin D1 protein levels. Moreover, miR-34 can upregulate p21, which inhibits CDK and blocks DNA synthesis, causing cell cycle arrest [50,148]. MiR-34a causes G1-phase arrest, reduces cell development, and promotes cell death in HepG2 cells by suppressing the production of cyclin D1, CDK4, and CDK6 [221]. Prostate [222], breast [223], ovarian [223], bladder [224], stomach [159], and laryngeal squamous cell carcinomas all show low expression of miR-101 (LSCC) [160]. MiR-101 increases the expression of the tumor suppressors p14, p16, p21, and p27 while suppressing the expression of c-Myc, CDK2, CDK4, CDK6, CDK8, cyclin D2, cyclin D3, and cyclin E2. MiR-9 can suppress the proliferation of malignant tumor cells by downregulating CDK6 and cyclin D1, causing cell cycle arrest in the G0/G1 phase [161]. By specifically suppressing the CDK6/cyclin D1/p21/Waf1 pathway, miR-218 can play a significant role in limiting the growth of glioma cells [164,165]. These miRNAs exhibit anti-proliferative properties, function as cancer tumor suppressors, and are frequently repressed in malignancies in a variety of ways. Nc886 [200], a regulatory ncRNA of intermediate length (101 nucleotides), is regarded as a precursor miRNA due to its length. DNA methylation in the promoter of nc886 hinders the expression of this gene in malignant tumors. In contrast to miRNAs and nuclear lncRNAs, which attach to complementary paired RNA or DNA targets. Nc886 binds to target proteins to influence their activity, which in turn regulates the expression of genes. It has been demonstrated that nc886 silencing results in decreased expression of intracellular CDK inhibitors such as CDKN2A and CDKN2C and increased expression of CDK4.

The E2F factor, which is necessary for the G1 phase to transition into the S phase, is released when the RB is phosphorylated by the CDK/cyclin complex. The E2F family is essential for cell cycle and apoptosis regulation. The RB protein family strictly regulates their activity, but transcription, post-translational modification, and protein stability also play a role in controlling how these factors are activated. MiRNAs can target E2F and control both its expression and function. First discovered by O’Donnell et al. [171], c-Myc-regulated miRNAs (miR-17-5p and miR-20a) can control the expression of E2F1 [170]. Several miRNAs, including miR-125b [154], miR-210 [166], miR-195 [127], miR-17-5p, and miR-20a [170] have been found to negatively regulate the expression of E2Fs. The miR-17-92 cluster modulates the translation of E2Fs mRNA [171]. It was discovered that proliferative cyclin D1, E2F1, and anti-proliferative p21, PTEN, RB1, RBL1 (p107), and RBL2 (p130) are common targets of miR-17, miR-20a, and miR-106b [172]. These miRNAs alter USSC cell cycle progression because they not only directly target proteins that control the cell cycle but also encourage cell division and control G1/S transitions by boosting the intracellular activity of E2F transcription factors. In prostate and stomach cancer, miR-330 and miR-331-3p inhibit E2F1 activity, causing cell cycle arrest [181,182].

**Figure 5:**
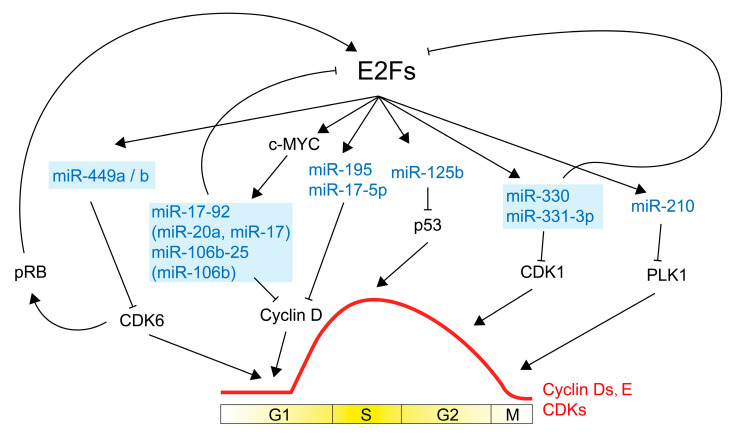
Schema showing E2Fs’ transcription on miRNAs and the feedback loop between miRNAs and E2Fs during the cell cycle. MiRNAs regulated by E2Fs are anti-proliferation, and they are in blue. MiR-17-92 and miR-106b-25 clusters are upregulated by c-MYC and inhibit E2F activity. MiR-449 downregulates CDK6, then reduces phosphating RB and suppresses E2Fs. MiR-330 and miR-331-3p inhibit E2F activity, causing cell cycle arrest. Blue squares show miRNAs that regulate E2Fs. S, DNA synthesis; M, mitosis; G1 and G2, index transition phase; G0, index quiescent status. Black lines end with arrow meaning promotion, black lines end with bar meaning inhibition.

Between E2F and miRNA, there is also automatic feedback control (Figure 5). E2F1 has been shown to directly transcribe miR-17-92 and miR-106b-25, which in turn induces their expression [225,226,227]. As a result, the expression of E2F1 is suppressed. MiR-17-92 and miR-106b-25 both target the 3′UTR of E2F1. The miR-17-92 cluster member miR-20a controls translation by interacting with the 3′-UTR region of the E2F2 and E2F3 mRNAs [146]. Moreover, endogenous E2F1-3 can bind directly to the miR-17-92 cluster’s promoter and trigger transcription, indicating the possibility of an auto-regulated feedback loop between the miR-17-92 cluster and E2Fs. The automated regulation of E2F1-3 and miR-20a is crucial for mediating cell proliferation and death and can effectively avoid the aberrant aggregation of E2Fs. In conjunction with E2F, these miRNAs create a negative feedback loop that gives organisms homeostatic security. Furthermore, E2F1-induced miRNAs such as miR-449a and miR-449b can negatively control RB phosphorylation by specifically targeting and inhibiting CDK6, thereby suppressing E2F1 activity and cell cycle progression [183,228]. This is an indirect way of describing how E2F1-activated miR-449 negatively regulates E2F1 activity.

Moreover, miRNAs contribute to the later phases of the cell cycle (Figure 4 and Table 2). Following the G1/S transition, complexes of CDK1 and cyclin A or cyclin B are primarily responsible for controlling the rest of the cell cycle. Let-7 is a negative regulator of the expression of several cyclins, including CDK4/6, cyclin D, cyclin A, and cyclin B [136,137,138,139]. The expression of cyclin A or cyclin B can be inhibited by miR-125b and miR-24 [154,229]. Some miRNAs may be engaged in different cell cycle phases and can control the expression of nearly all CDKs. Hydbring et al. [230] studied a wide range of cancer cells and discovered that a class of independent miRNAs (including miR-193b, miR-193a-3p, miR-195-5p, miR-214-5p, and miR890) targeted almost all cyclins and CDKs. These miRNAs significantly reduce cancer cell proliferation.

Although they report less frequently, miRNAs can also control the mitotic process. Polo-like kinase (PLK1) is a crucial mitosis regulator [231]. PLK1 phosphorylates CDC25C, which activates the CDK1/cyclin B1 complex and causes it to translocate into the nucleus to initiate mitosis in the cell cycle. PLK1 expression is negatively regulated by miR-100, which targets PLK1 mRNA [232]. Moreover, paclitaxel therapy markedly decreases miR-100 level and enhances mRNA stability and transcription of β-tubulin I, IIA, IIB, and V, all of which are attributed to G2/M block [128]. MiR-100 targets and inhibits MTMR3, further suppressing p27, which is critical for the proliferation of breast cancer cells [129]. Downregulation of MiR-100 can activate p27, causing G2/M cell cycle arrest and apoptosis as a result. In osteosarcoma, miR-223 can act as a tumor suppressor by upregulating the expression of p21 and p27, increasing RB phosphorylation, and causing G1 phase arrest [213]. In breast cancer, miR-223 elevates p27 expression. On the one hand, p27 directly improves the stability of mature miR-223 [214]. On the other hand, p27 improves E2F1’s control of miR-223’s promoter activity. In addition, p27 increases miR-223 expression in two ways. The mutual control of p27 and miR-223 is important for cell cycle arrest and the abolishment of contact inhibition. More research is still needed to determine how functionally important miRNAs are in controlling mitosis.

The INK4 and Cip/Kip families are also regulated by miRNAs, either directly or indirectly (Figure 4 and Table 2). P16 is regulated by miR-31 [187,188]. MiR-106b and the miR-17-92 cluster (including six miRNA members: miR-17, miR-18a, miR-19a, miR-20a, miR-19b, and miR-92a-1) directly target p21 [178,179,180,233,234]. MiR-24 targets and inhibits p21, p16, and p27 [155,235,236]. In small-cell lung cancer (NSCLC) cells A549 and H1298, miR-512-5p targets and suppresses p21, which causes apoptosis and reduces glycolysis [189]. MiR-370 inhibits p27 and p21 while increasing cyclin D1 to promote the G1/S phase transition [190,237]. MiR-370 plays a crucial role in the malignant progression of prostate, lung, and bladder cancers. MiR-212 enhances p21 and p27 expression by decreasing retinoblastoma binding protein 2 (RBP2), which delays the G1/S phase transition and indirectly decreases proliferation [191,192]. MiR-196a suppresses cell cycle progression by inhibiting p27 expression and cyclin E/CDK2 activity [193,238]. Skp2’s 3′-UTR mRNA binds to miR-3163, which prevents Skp2 from being translated [194]. Skp2, a part of the E3 ubiquitin ligase SCF, especially encourages the ubiquitination degradation of the p27 and supports the development of cancer cells in various cancers. By inhibiting Skp2, miR-3163 increases the stability of p27 and slows the development of cancer cells. MiR-221/222 inhibits p27 and p57 and activates CDK2, both of which are factors in tumor progression [195,196,197,198,239]. MiR-222 increases p57 expression and accelerates the G1 to S phase transition [199]. MiR-222 also increases Capan-2 pancreatic cancer cells’ ability to survive and proliferate. A novel approach to the treatment of pancreatic cancer may involve targeting miR-222.

In the aging model, miR-17, miR-19b, miR-106a, and miR-20a were also found to be related to the regulation of the transcription level of target genes, especially p21 [143]. These miRNAs may serve as new markers of human aging. In INK4/ARF locations, polycomb repressive complexes (PRC1 and PRC2) are epigenetic regulators that control aging. MiR-9 suppresses the expression of CBX7, a component of PRC1. In turn, CBX7 suppresses miR-9, resulting in a negative feedback loop [162,163]. The miR-9/CBX7 feedback loop regulates aging by inducing the production of p16. MiR-124a can bind to the 3′-UTR region of MCP1′ (monocyte chemoattractant protein 1) and CDK2′ mRNAs and specifically inhibit their activity [150,151]. MiR-124a significantly inhibits CDK2 expression in synovial cells of patients with rheumatoid arthritis (RA), thereby inhibiting cell proliferation and arresting the cell cycle in the G1 phase. The stability of miR-17, miR-20-5p, and miR-106-5p is reduced quickly throughout the process of oxidative stress, limiting DNA synthesis and blocking the G1/S transition [177]. Overexpression of miR-20b-5p/miR-106a-5p directly inhibits p21, cyclin D1, and E2F1, which can reverse growth retardation by increasing DNA synthesis and G1/S transition.

In addition, various cell cycle conditions may alter miRNA expression and stability. The first ncRNA to be reported to be regulated by cyclin is miR-17/20, which is bound to cyclin D1 [240]. Cyclin D1 controls miRNA maturation. The RNase III endoribonuclease Dicer plays a crucial regulatory role in the development of miRNA by cleaving long-stranded RNA or pre-miRNA with stem ring structure into mature miRNA. According to studies [241], cyclin D1 regulates the generation of miRNAs by promoting the expression of the Dicer both in vivo and in vitro using targeted transcription mechanisms. Cyclin D1 can control both the processing of many miRNAs and the expression of individual miRNAs by binding to regulatory areas and stimulating the transcription of the Dicer enzyme.

## 5. NcRNAs and CDKI Treatment

### 5.1. CDKIs and Cancer Treatment

CDK inhibitors were developed to impede the development of cancer cells. Currently, there are three generations. Although they have excellent anticancer efficacy in preclinical and clinical studies, generation 1 and 2 CDKIs have not received much therapeutic attention in the treatment of cancer patients due to their low selectivity and severe toxicity [242]. By selectively targeting CDK4 and CDK6, the third generation of CDKIs has achieved outstanding success in clinical applications, significantly reducing the development of several malignant tumors (Table S1). Nowadays, drugs that precisely target CDK4/6 activity are used extensively in cancer therapy. Due to their efficacy in treating breast cancer, more than ten CDK inhibitors are presently undergoing clinical trials (Table S1).

Oral CDK inhibitors with specific chemical structures that bind to CDK4 and CDK6 include palbociclib [243,244], ribociclib [245,246], and abemaciclib [247]. They cause G1-phase arrest and inhibit RB phosphorylation [7]. Palbociclib is the first CDK4/6-cyclin Ds inhibitors FDA-approved for postmenopausal HR-positive and HER2-negative metastatic breast cancer [243]. The anticancer effects of palbociclib have been reported in neuroblastoma [248], non-small cell lung cancer [249], and breast cancer [250]. Palbociclib suppresses RB phosphorylation, downregulates E2F, causes G1-phase cell cycle arrest, and inhibits tumor growth, according to in vitro and in vivo studies [251,252]. Palbociclib can also make medulloblastoma cells more responsive to chemotherapy and ionizing radiation therapy [253]. In RB-positive cancer cells, ribociclib causes the dephosphorylation of pRB, which results in G1-phase arrest [248]. In RB-positive tumors such as ER-positive breast cancer and neuroblastoma, ribociclib’s antitumor effects have been examined [248,254]. Ribociclib dramatically reduces cell proliferation and enhanced apoptosis in ER-a breast cancer cell lines [255]. In both vitro and vivo experiments, abemaciclib improves the effects of chemotherapeutic medicines on cells overexpressing the ATP Binding Cassette Subfamily B Member 1 (ABCB1) and ATP Binding Cassette Subfamily G Member 2 (ABCG2) [256]. In breast cancer and other cancers, its anticancer properties have been studied. Ribociblib and abemaciclib have also been approved for the combination endocrine therapy of advanced HR-positive and HER2-negative breast cancer in phase III clinical trials [247,257,258,259]. Currently, in the preclinical or early clinical stages, CDK4/6 inhibitors are promising medications for the treatment of malignant cancers. In comparison to endocrine therapy alone, the combination utility of CDK4/6 inhibitors and endocrine therapy in the first-line treatment of advanced breast cancer prolonged PFS in phase III clinical trial MONARCH 3 [260,261,262].

CDKIs have been known to cause acquired resistance through several mechanisms [263,264]. Approximately 10% of patients will initially develop resistance to CDK4/6 inhibition, and a growing number will eventually lose their effectiveness [264]. To pinpoint non-responders to CDK4/6 inhibition and to individualize therapy, new biomarkers are required. Cell cycle regulators, including p16, CDK6, CCNE1/2, CDK2, CDK7, and E2F, are crucial in developing resistance to CDK4/6 inhibition [255,265,266]. The CDK inhibition palbociclib becomes partially resistant when pRB is inhibited [267,268,269]. P16 gene-deficient animals show acquired resistance to CDK4/6 drugs in preclinical studies [270]. Patients with high P16 expression and decreased RB expression typically developed resistance to CDK4/6 inhibitors and were ineligible for CDK4/6 inhibitor clinical trials [271]. Acquired resistance to CDK4/6 inhibitors may be brought on by acquired RB mutations, cyclin E amplification, CDK6 amplification, or inhibition of CDK2 inhibitors (such as p27 or p21) [271,272,273,274,275].

In addition, CDKIs tend to induce dose-related neutropenia with rare neutropenic fever. Ribociclib is associated with prolonged QT and therefore requires ECG monitoring. Abemaciclib is more likely to cause gastrointestinal toxicity, which manifests as diarrhea. Abemaciclib has a higher incidence of fatigue [276]. The main adverse event in palbociclib is neutropenia, and other adverse events include fatigue, diarrhea, nausea, dyspnea, and joint pain [277].

### 5.2. LncRNA and CDKI Clinical Efficiency

There are few reports and only preclinical stage studies on the reduced or enhanced effect of lncRNA on CDKIs (Table 3). LncRNA TROJAN is significantly expressed in TNBC and promotes the transfer potential of TNBC, which has detrimental effects [278]. TROJAN is associated with a decreased disease-free survival rate in patients with ER+ breast cancer [279]. Moreover, TROJAN can accelerate the growth and development of ER+ breast cancer by controlling the cell cycle during G1/S conversion. Downregulation of TROJAN causes ER+ breast cancer cells to be arrested in G1, resulting in a decrease in cyclin E1/2 and an increase in p21 and p27. TROJAN can bind to NKRF, an NF-B pathway repressor, blocking it from interacting with RELA, an NF-B pathway transcriptional activator. The TROJAN-NKRF complex can control the transcriptional frequency of CDK2 expression and prevent NKRF from binding to the CDK2 promoter, making tumor cells resistant to CDKIs [255,280]. Increased effectiveness of CDK4/6 inhibitors can be achieved by inhibiting TROJAN, which can also limit CDK2 expression and prevent non-classical CDK2-mediated entry into the S phase. Combining TROJAN inhibition with CDK4/6 inhibitors has been shown to dramatically enhance the anticancer activity of CDK4/6 inhibitors [100]. When treated with CDK4/6 inhibitors, CDK2 activation is a crucial bypass mechanism for cell cycle advancement [280].

LncRNA SNHG15 performs a cancer-promoting role by encouraging proliferation and metastasis in glioblastoma (GBM), breast cancer, lung cancer, and liver cancer [286,287,288]. SNHG15 increased the overall survival of HCC and has a favorable correlation with TNM staging [288]. Increased SNHG15 is linked to a significantly higher risk of GBM and poorer overall survival in GBM patients [289,290]. SNHG15 activates CDK6 by suppressing the tumor suppressor miR-627-5p. In tumor-bearing mice, palbociclib significantly increased the expression of miR-627-5p while decreasing the expression of SNHG15 and CDK6 [101].

LncRNA maternally expressed gene 3 (MEG3, mouse homolog Gtl2) has been shown to act as a tumor suppressor in a variety of human cancer cell lines [291,292,293,294]. By directly interacting with chromatin, MGE3 inhibits the TGF-β pathway or activates p53 to inhibit cell proliferation [292,295,296]. The CDK4/6 inhibitor palbociclib activates pRB in lung cancer cells (A549 and SK-MS-1), and pRB particularly encourages MEG3 expression [281]. This demonstrates that palbociclib or other CDKIs can be used to target a range of malignancies that depend on the activation of the MEG3 regulatory pathway. The decline in cell proliferation caused by palbociclib can be partially restored by MEG3 expression suppression. Palbociclib’s therapeutic effectiveness may therefore be reduced by the tumor cells’ low MEG3 expression. Palbociclib, a CKD4/6 inhibitor, increases MEG3 expression in tumor cells, indicating that it may be effective against tumor types whose growth is regulated by MEG3. Furthermore, CDK4/6 inhibitors may be used in combination with other medications now on the market. For example, lower MEG3 expression is associated with cisplatin resistance in NSCLC [297]. MEG3 high expression caused by CDK4/6 inhibitors may be beneficial for platinum-based therapy. More studies are required to determine whether palbociclib and cisplatin are effective when combined.

### 5.3. MiRNA and CDKI Clinical Efficiency

MiRNAs can affect CDKIs negatively or positively because of the intricate relationships between them and cell cycle progression. MiRNAs are a valuable alternative to or combination therapy for CDK4/6 CDK4/6 inhibitors and can be used as biomarkers to predict the therapeutic efficacy of CDK4/6 CDK4/6 inhibitors. MiR-497 inhibits the growth of anaplastic large cell lymphoma (ALCL) by negatively regulating the expression of the cell cycle regulatory genes CCNE1, CDC25A, CDK6, and E2F3 [204,205]. NPM-ALK (+) ALCL cells are susceptible to palbociclib treatment in a CDK6-dependent manner. Cell cycle arrest in the G1/S phase is caused by Nc886’s downregulation of CDK4 expression and upregulation of CKIs (such as p16 and p18) [200,201]. Nc886-silenced cells have increased G1 to S phase activity, rapid cell division, and are more sensitive to palbociclib. Hence, in cancer patients, nc886 can be utilized as a predictor of medication reactivity. In prostate cancer, miR-193b suppresses cyclin D1 expression [206,207,208,209]. Overexpression of miR-193b in prostate cancer cells prevents cells from progressing from the S phase to the G2/M phase. In contrast, prostate cancer cells with high miR-193b expression become resistant to CDK4/6 inhibitors. Reduced expression of miR-193b in prostate cancer cells results in upregulation of cyclin D1 and greater susceptibility to palbociclib therapy. Oral squamous cell carcinoma (OSCC) has decreased miR-9 expression [161]. It was discovered that miR-9 negatively regulates the expression of CDK6 and cyclin D1 and that miR-9 overexpression inhibits cell proliferation, reduces the capacity of colonies to form, and causes cell cycle arrest in the G0/G1 phase. MiR-9’s ability to inhibit cell proliferation in OSCC may serve as a theoretical foundation for the creation of small compounds that target CDK4/6.

Because CDKIs have achieved positive outcomes in the treatment of breast cancer, many research studies have shown the impact of miRNA on the sensitivity of CDKIs in breast cancer. MiR-3613-3p stops TNBC cells from proliferating, which causes a cell cycle arrest in the G0/G1 phase [282]. MiR-3613-3p increases senescence, which increases the susceptibility of breast cancer cells to palbociclib. Palbociclib-sensitive breast cancer cells produce miR-29b-3p at higher levels, but if the miR-29b-3p expression is decreased, the cells may become resistant to treatment [202,203]. Particularly in the luminal subtype of malignant breast cancers, miR-223 expression is downregulated [283]. Lower levels of miR-223 are related to a worse prognosis and reduced overall survival. In luminal breast cancer cells, downregulation of miR-223 expression is associated with palbociclib resistance. In addition, palbociclib resistance is boosted by suppressing miR-223 in HER2-positive breast cancer cells. In contrast to CDK4/6 inhibitor-sensitive cancers, miR-432-5p expression is higher in ER-positive tumors with resistance [285]. MiR-432-5p increases CDK6 expression and is attributed to palbociclib or ribociclib resistance. Loss of CDK6 causes resistant cells to regain their sensitivity to palbociclib.

MiRNAs influence the sensitivity of CDK4/6 inhibitors in addition to other tumor cell processes such as metastasis and metabolism, which may suggest novel medication combinations. By inhibiting CDK6 and creating cell cycle stasis in the G1 phase, miR-29b-3p inhibits proliferation via limiting cell motility and the epithelial–mesenchymal transition (EMT) [202,203]. In metastatic melanoma, miR-200a is down-expressed, which negatively regulates CDK6 expression and slows down G1/S checkpoint cell cycle progression [210]. In metastatic melanomas, high miR-200a and low CDK6 expression are associated with palbociclib resistance, whereas low miR-200a and high CDK6 expression make metastatic melanomas more responsive to CDK4/6 inhibitors. By blocking CDK4/6, miR-126 controls glycolysis and the advancement of the M phase, and it has a clear anti-proliferative effect on breast cancer cells [284]. In luminal and HER2-positive breast cancer cells, the combination of overexpressed miR-126 and the CDK4/6 inhibition ribociclib exhibits potent antitumor effects that are far more effective than those of either treatment alone. MiR-126 dramatically increases the susceptibility of HER2-positive breast cancer cell lines to ribociclib.

The response of tumor cells to different CDK4/6 inhibitors may be influenced by miRNAs (Table 3). A single transcript from the miR17HG gene on human chromosome 13 is processed into the miR-17-92 cluster [173,298]. A total of six mature miRNAs are present in the Mir-17-92 cluster (miR-17, miR-18a, miR-19a, miR-20a, miR-19b, and miR-92). Atypical teratoid rhabdomyoma (ATRT) has been reported to respond therapeutically to palbociclib when miR-19a, miR-17, and miR-20a block cyclin D1 expression. Both ATRT cell lines and mice with ATRT tumors show greater sensitivity to CDK4/6 inhibitors. Moreover, it was discovered that the miR-17-92 family is upregulated in glioblastoma neuropathy (GBM), increasing the sensitivity of GBM stem-like cell lines to palbociclib and ribociclib. MiR-17-19b significantly shortens the time required for the emergence of mixed lymphocytic lymphoma (MLL) by suppressing the expression of p21 [174,175]. By regulating p21 expression, miRNAs influence leukemia stem cell (LSC) self-renewal. MiR-19a or miR-19b controls cell cycle progression and inhibits Wee1 in leukemia [176]. In the G2/M phase of head and neck squamous cell carcinoma (HNSCC) cells, MiR-17-92 works as a transcription factor. MiR-17-92 negatively influences the mRNA and protein expression of cyclin G2 by targeting its 3′-UTR [298].

MiRNAs are thought to play a significant role in the effectiveness of CDK4/6 inhibitors and are crucial in the control of cancer-related pathways. To help doctors make treatment decisions, miRNA expression profiles may be employed as predictive biomarkers for the effectiveness of CDKIs.

## 6. Perspective and Conclusions

NcRNAs participate in different phases of the cell cycle by focusing on specific signaling pathways and related elements. NcRNAs can be valuable choices for therapeutic drugs or therapeutic targets since they can target many altered mRNAs in illness circumstances. Unrestricted proliferation brought on by an unchecked cell cycle is a hallmark of cancer [299,300,301]. CDKs play a vital role in regulating the cell cycle, so inhibitors that specifically target different CDKs have been developed to stop the proliferation of cancer cells. We postulate that because ncRNAs and the cell cycle are so closely related, dysregulated ncRNAs may provide strategies for increasing CDKIs’ therapeutic benefits in cancer. However, further investigation is required to comprehend the importance of ncRNAs in the cell cycle and the therapeutic function of CDKIs as well as to outline suitable methods for enhancing the clinical use of CDKIs.

In preclinical and clinical research, a large number of CDK4/6 inhibitors have been found to limit the growth of many malignancies, particularly breast cancer. Clinical evidence from research on the condition suggests that CDK4/6 inhibitors may be effective as a single therapy for advanced breast cancer. Moreover, CDK4/6 inhibitors can be combined with chemotherapy or endocrine treatments to enhance their effectiveness [243,247,257,258]. Even though taking CDKIs has been linked to some adverse effects, including neutropenia, this is primarily due to dose restrictions. New CDKIs with lower toxicity should be investigated, or CDKIs with different adverse features should be created to lessen side effects such as myelosuppression. More importantly, alternative molecular processes continue to drive the cell cycle in the absence of the CDK mechanism, leading to tumor resistance to CDK4/6 inhibitors and long-term growth. Thus, consideration must be given to the design of inhibitors that target various cyclin/CDK domains.

It is still unclear exactly how ncRNAs control the cell cycle, since the regulation of the cell cycle by ncRNAs is more complex. Certain ncRNAs influence proliferation not just by targeting a single cell cycle regulator but also by targeting both positive and negative cell cycle regulators, such as members of the mir-17-92 cluster [219,233]. For instance, miR-369-3p inhibits the proliferation of liver cancer cells by positively regulating the production of p21 and negatively regulating the expression of cyclin D1 in liver cancer [302]. MiR-369-3p suppresses the stability of p53 in ovarian granulosa cells and decreases apoptosis in ovarian granulosa cells (OGCs) [303]. Moreover, the stability of miRNAs may fluctuate during a particular cell cycle, and miRNA regulation may be cell cycle-dependent. Depending on the stage in the cell cycle, miRNAs may be able to either up or downregulate translation. For example, members of the miR-369-3p or let-7 family may promote translation in stationary cells and suppress translation during cell cycle/proliferation [304].

Because the links between ncRNAs and mRNAs or proteins constitute a complicated network, it is vital to investigate the interaction between ncRNAs and targets in specific settings. Many variations exist in different studies because of differences in tumor types, separation procedures, detection methods, and sample processing. It is possible that different types of cells do not exhibit the same kinds of interactions between ncRNAs and their targets. As a result, it is necessary to discuss the relationship between ncRNAs and their targets in the context of certain cellular circumstances. Many ncRNA targets are still unknown due to the lack of strong evidence-based ways to prove the direct targeting and control of ncRNAs. Proteomics is a valuable method for discovering whole-cell target genes and examining the influence of miRNAs on proteins. Most existing links between ncRNAs and the cell cycle remain undiscovered [305,306].

According to some preclinical research, ncRNAs influence how cancer cells respond to CDK4/6 inhibitors [207]. By modifying the expression of downstream targets (CDK4/6 and cyclin Ds) or by controlling associated signaling pathways, some ncRNAs can make cancer cells resistant to CDK4/6. Some ncRNAs suppress the production of cyclin Ds or interfere with the expression of cell cycle regulatory genes, making cancer cells more sensitive to CDK4/6 inhibitors. Further consideration should focus on the function of ncRNAs in CDK4/6 inhibition management. In addition to changing ncRNA expression and function, DNA damage can alter the cell cycle components, and eventually slow down the cell cycle process. The DNA damage response is associated with some lncRNAs [281,307]. At the DNA damage checkpoint, lncRNAs cause CDK inactivation, cell cycle arrest, or apoptosis, mediating the non-classical pathway of the DNA damage response [96,97]. DNA damage is the primary mechanism by which most platinum-based chemotherapy medicines treat tumors. More research is required to determine if changes in intracellular ncRNAs brought on by DNA damage treatments would increase or lessen the effects of CDKI therapy. The degree of ncRNA expression also affects the susceptibility of cancer cells to chemotherapeutic treatments [308,309,310]. As a result, the combination of CDK4/6 inhibitors and other active therapeutic medications may be effective. Significant differences in ncRNA expression patterns in cancer cells are related to an enhanced or decreased response to CDK4/6 inhibitors [311,312]. However, there is currently insufficient clinical evidence to support this connection.

Direct targeting of ncRNAs or altering intracellular levels of ncRNAs may both be effective therapeutic approaches. Although several ncRNA mimics and chemically modified anti-ncRNAs have been designed, delivering these compounds to the target location and establishing their effective concentrations remain challenging problems [313,314,315,316]. Several vectors have been created, such as cell-specific absorption of oligonucleotides based on lipids, polymers, metal nanoparticles, molecular peptides, etc. The therapeutic applicability of these vectors, however, requires more study. Challenges with whole-body administration, plasma degradation, and difficult-to-deliver targets (such as cardiovascular tissues and the central nervous system) are just a few of the clinical applications that still need to be explored.

In conclusion, the use of ncRNAs in treatment helps us comprehend the link between ncRNAs and the cell cycle. This study has concentrated on the influence of ncRNAs on the cell cycle as well as the effect of the cell cycle on the composition and function of ncRNAs. Further research is required to determine how ncRNAs affect CDK4/6 inhibitors and to generate novel treatment targets and effectiveness indicators based on this connection.

## Figures and Tables

**Table 1:** LncRNA associated in the cell cycle control.

LncRNA	Target in Cell Cycle (Phase)	Deregulation in Cancer	References
MEG3	Promotion of p27 by miR-3163/Skp2 mRNA translation.	Downregulated in BLCA, BRCA, CCA, COCA, OVCA. Upregulated in small cell lung cancer.	[88]
LINC-PINT	Suppression of CDK1 and cyclin A2 with PRC2 and EZH2 (G2/M).	Downregulated in HCC, BLCA, BRCA, MM, T and B-ALL. Upregulated in COCA.	[75,76]
HEIH	Suppression of p15, p16, p21 and p57 with EZH2 (G0/G1).	Upregulated in HCC. Downregulated in BRCA, CRCA, OVCA, PRCA.	[77]
ANRIL	Suppression of p21, p14, p15, p16 upregulated cyclin G1 by targeting miR-181a-5p (G0/G1, G2/M).	Upregulated in glioma, LSCC, GC, EOC, HNSC, NSCLC, ESCC, CRCA.	[78,79,80]
LINC00630	Promotion of CDK2 transcription (S).	Upregulated in HCC, CRCA.	[84]
ZFAS1	Promotion of p21 and p27 expression; destabilization of p53; promotion of CDK1/cyclin B1 complex by interaction (G1/S).	Upregulated in CRCA, BLCA, GC, HCC, PRCA. Downregulated in OVCA, basal-like BRCA.	[94]
TMPO-AS1	Promotion of CDK1. Promotion proliferation by sponging miR-326 and targeted by E2F1 (G0/G1, G2/M).	Upregulated in BLCA, basal-like BRCA, CCA, CRCA, LUCA, PRCA, HCC, OVCA.	[82]
ARAP1-AS1	Promotion of cyclin D1 (G0/G1).	Upregulated in LUCA.	[85]
CADM1-AS1	Promotion of p15, p21, p27. Suppression of cyclin Ds, cyclin Es, CDK2, CDK4, CDK6 (G0/G1).	Downregulated in HCC.	[95]
TPT1-AS1	Promotion of CDK4, cyclin D1, suppression of p21 (G1/S).	Upregulated in GC, HCC. Downregulated in BLCA, BRCA, LUCA, OVCA, PRCA.	[89]
ABHD11-AS1	Promotion of Cyclin D1, cyclin E1, CDK1, CDK2, CDK4. Suppression of p16 (G1/S).	Upregulated in EC, PCA, GC, LUCA. Downregulated in CRCA.	[90,91,92,93]
CCDC144NL-AS1	Promotion of CDK1, CKD2 and CDK4 (G0-1/S) through miR-940/WDR5.	Upregulated in CRCA, HCC. Downregulated in LUCA.	[99]
LINC00261	Co-regulation with CDK1 (G0/G1; G2/M).	Downregulated in choriocarcinoma, HCC, BRCA, PRCA, CRCA, GC, LUCA.	[83]
Gadd7	Suppression of CDK6 mRNA degradation (G1/S).	Induced by DNA damage and oxidative stress.	[96,97]
ALMS1- IT1	ALMS1-IT1/AVL9 promote CDK pathway.	Upregulated in small cell lung cancer, CCA, CRCA, OVCA. Downregulated in LUCA.	[86]
XIST	Promotion of cyclin D1, CDK1 and p53, suppression of p21 (G1). Suppression of Wee1 (G2).	Upregulated in PCA, CRCA, ESCC. Downregulated in OVCA, NSCLC, HCC, BRCA, BLCA.	[63,98]
ENST00000512916	Promotion of CDK2/4/6 and cyclin D1 (G1).	Upregulated in AB.	[87]
TROJAN	Promotion of CDK2.	Upregulated in ER-positive BRCA.	[100]
SNHG15	Promotion of CDK6 by miR-627 sponging.	Upregulated in BLCA, TNBC, CRCA, GC, LUCA, PRCA.	[101]

Abbreviations: BRCA, breast cancer; CCA, cervical cancer; COCA, colon cancer; BLCA, bladder cancer; HCC, hepatocellular carcinoma; CRCA, colorectal cancer; OVCA, ovarian cancer; PRCA, prostate cancer; T, T cell; B, B cell; ALL, acute lymphocytic leukemia; GC, gastric cancer; TNBC, triple-negative breast cancer; LSCC, laryngeal squamous cell cancer; EOC, epithelial ovarian cancer; HNSC, head and neck squamous cell carcinoma; NSCLC, non-small cell lung cancer; ESCC, esophageal squamous cell carcinoma; LUCA, lung cancer; EC, endometrial carcinoma; PCA, pancreatic cancer; MM, malignant melanoma; AB, ameloblastoma. S, DNA synthesis; M, mitosis; G1 and G2, index transition phase; G0, index quiescent status.

**Table 2:** MiRNAs associated with cell cycle control.

miRNA	Target in Cell Cycle (Phase)	Deregulation in Cancer	References
miR-195	Suppression of CDK6, cyclin E1, CDK4, cyclin D1, cyclin D3 (G1/S).	Downregulated in NSCLC, GC, HCC, BLCA. Upregulated in BRCA, CLL, ACA.	[126,127]
miR-100	Targeting and suppressing PLK1; suppression of reduced β-tubulin I, IIA, IIB and V mRNA; suppression of p27 (G2/M).	Upregulated in BRCA, PCA. Downregulated in PRCA, OVCA, BLCA.	[128,129]
miR-365	Suppression cyclin D1 and CDC25A, promotion of p27 (G1/S).	Downregulated in NSCLC, BRCA, OVCA.	[130,131]
miR-15a	Suppression of CDK1, CDK2, CDK6, cyclin D1, cyclin D2 and cyclin E1 (G1/S).	Downregulated in HCC, BRCA, OS, NSCLC, PCA, PRCA. Upregulated in CLL.	[132,133,134,135]
miR-16	Suppression of CDK1, CDK2, CDK6, cyclin D1, cyclin D2 and cyclin E1 (G1/S).	Downregulated in PRCA, GC, LUCA. Upregulated in PCA, HCC.	[133,134,135]
Let-7	Suppression of CDK4, Cyclin D1, cyclin D3, Cyclin A and Cyclin B translation (G1).	Downregulation in OVCA, LUCA, NSCLC, BRCA, CRCA, PRCA, HNSC.	[136,137,138,139]
miR-7	Suppression of p53, CDC42, Cyclin D, cyclin E1 (G0-1/S).	Downregulated in BRCA, CCA, MG, HCC. Upregulated in HNSC, LUCA.	[136,140,141]
miR-19	Suppression of Cyclin Ds, promotion of p21 (G1/S).	downregulated in MG.	[142,143]
miR-20a	Suppression of E2F2 and E2F3 translation; Suppression cyclin D1 mRNA transcription; Suppression of p21 (G1/S).	Upregulated in HCC, CRCA, PRCA, PCA, LUCA. Downregulated in BRCA, HCC.	[144,145,146]
miR-34	Suppression of Cyclin D1, CDK4, CDK6 translation; promotion of p21 (G1/S).	Downregulated in PCA, OVCA, NSCLC, HCC. Upregulated in CRCA.	[147,148]
miR-124a	Suppression CDK2, CDK6, cyclin D1, cyclin D2 translation (G1).	Downregulated in all cancer.	[149,150,151]
miR-125b	Suppression cyclin D1, CDK6, promotion of p21 (G1/S).	Downregulated in PRCA, LUCA, HCC, BRCA, CCA, CRCA. Upregulated in PCA, BLCA.	[152,153,154]
miR-24	Suppression of p27, p16, p21(G2/M).	Downregulated in PRCA, PTC. Upregulated in LUCA, PCA, CRCA.	[154,155]
miR-26a	Suppression of cyclin D2, cyclin D3, cyclin E2, CDK4/6; promotion of p14.	Downregulated in PTC, LUCA, HCC, BRCA, BLCA.	[156,157,158]
miR-101	Suppression of CDK2, CDK4, CDK6, cyclin D2, cyclin D3 and cyclin E2; Promotion of p14, p16, p21 and p27 (G1).	Downregulated in LUCA, PRCA, HCC, LSCC, OVCA, BLCA.	[159,160]
miR-9	Suppression of CDK6, cyclin D1; promotion p16 (G0/G1).	Downregulated in HSCC.	[161,162,163]
miR-218	Suppression CDK6, cyclin D1; promotion of p14, p16.	Downregulated in MG, LUCA, CCA. Upregulated in PRCA.	[164,165]
miR-210	Suppression of CDK4, CDK6; promotion of p27 (G1/S).	Upregulated in PRCA, LUCA, PCA, HNSC, BRCA.	[166]
miR-17-5p	Suppression of cyclin D1; promotion of p21 (G2/M).	Upregulated in bladder cancer, HCC, BRCA, CRCA, LUCA.	[144,167,168,169]
miR-17-92 cluster (MIR17HG)	Suppression of E2F1, cyclin D1. Promotion of p21 (G1/S).	Upregulated in BL, CRCA, LUCA, BLCA, BRCA, PRCA, PCA, HCC.	[170,171,172,173,174,175,176]
miR-106a	Suppression of p21, cyclin D1 (G1/S).	Upregulated in LUCA, HCC, PCA, PRCA, CRCA.	[143,177]
miR-106b	Suppression of cyclin D1, E2F1, p21 (G1/S).	upregulated in HCC, GC, CRCA. Downregulated in OVCA.	[172,178,179,180]
miR-330	Suppression of E2F1.	Downregulated in OSCC, PRCA.	[181]
miR-331-3p	Suppression of E2F1.	Downregulated in HNSC, OSCC. Upregulated in AML.	[182]
miR-449	Promotion of CDK6 and CDC25A; Suppression E2F1, E2F3, cyclin D1, cyclin A2.	Downregulated in PRCA.	[183,184]
miR-31	Suppression of CDK1, CDK4, CDK6; promotion of p21.	Upregulated in HNSC, CRCA, OSCC, LUCA. Downregulated in PRCA, BRCA, BLCA.	[185,186,187,188]
miR-512-5p	Suppression of p21.	Downregulated in GC.	[189]
miR-370	Promotion of p27 and p21 (G1/S).	Upregulated in PRCA. Downregulated in OSCC.	[190]
miR-212	Promotion of p21 and p27; suppression cyclin D3 (G1/S).	Upregulated in LUCA, PCA, HCC.	[191,192]
miR-196a	Suppression of p27.	Upregulated in BRCA, PDAC, ESCC, CRCA.	[193]
miR-3163	Promotion of p27 (G0/G1).	Downregulated in BRCA.	[88,194]
miR-221	Suppression of p27 and p57, promotion of CDK2 (G2/M).	Downregulated in PRCA, NSCLC. Upregulated in HCC, PCA, BLCA, CRCA, OVCA, BRCA, OSCC.	[195,196]
miR-222	Suppression of p27 (G1/S).	Downregulated in PRCA. Upregulated in PCA, NSCLC, HCC, GC.	[197,198,199]
Nc886	Promotion of CDKN2A and CDKN2C, suppression of CDK4 (G1/S).	Downregulated in ESCC, GC, PRCA.	[200,201]
miR-29b-3p	Suppression of CDK6 (G1).	Downregulated in PRCA, LUCA, HNSC, CLL, AML. Upregulated in BRCA, CRCA, PCA.	[202,203]
miR-497	Suppression of CDK6, E2F3 and cyclin E1 (G1).	Downregulated in PRCA, LUCA, CRCA, BRCA.	[204,205]
miR-193b	Suppression of cyclin D1 by targeting 3′UTR of mRNA (G1/S).	Downregulated in BRCA, PRCA, OS, MM.	[206,207,208,209]
miR-200	Suppression of CDK6 (G1).	Downregulated in CRCA, HCC, OV, BRCA, MM (all cancer)	[210]
miR-206	Suppression of CDK4, cyclin D1, cyclin C translation (G1).	Downregulated in BRCA, OVCA.	[211,212]
miR-223	Suppression of p21, p27 (G1). E2F1 binds to the miR-223 promoter and inhibits transcription.	Downregulated in AML, HCC, CLL. Upregulated in BLCA, PCA, CRCA, PRCA.	[213,214]

Abbreviations: BRCA, breast cancer; CCA, cervical cancer; BLCA, bladder cancer; HCC, hepatocellular carcinoma; CRCA, colorectal cancer; OVCA, ovarian cancer; PRCA, prostate cancer; GC, gastric cancer; LSCC, laryngeal squamous cell cancer; HNSC, head and neck squamous cell carcinoma; NSCLC, non-small cell lung cancer; ESCC, esophageal squamous cell carcinoma; LUCA, lung cancer; PCA, pancreatic cancer; MM, malignant melanoma; AML, acute myeloid leukemia; CLL, chronic lymphocytic leukemia; OS, osteosarcoma; OSCC, oral squamous cell carcinoma; BL, Burkitt lymphoma; MG, malignant glioma; PTC, papillary thyroid carcinoma; ACA, adrenocortical adenomas. S, DNA synthesis; M, mitosis; G1 and G2, index transition phase; G0, index quiescent status.

**Table 3:** NcRNAs associated with tumor sensitivity to CDK4/6 inhibitors.

ncRNA	Tumor Type	CDKIs	Sample	Function and Mechanism	References
MEG3	LC	Palbociclib	cell lines	Palbociclib increase MEG3 expression. Silencing MEG3 expression rescue palbociclib-mediated decrease in cell proliferation.	[281]
TROJAN	ER-positive BC, TNBC	Palbociclib	cell lines	TROJAN inhibit NKRF/RELA and upregulate CDK2 expression, which promote cell proliferation and resistance to a CDK4/6 inhibition in ER+ breast cancer.	[100]
SNHG15	GBM, BC, LC, HCC	Palbociclib	cell lines	SNHG15 upregulated CDK6 by sponging inhibit miR-627-5p. palbociclib treatment increased miR-627-5p expression and reduced SNHG15 and CDK6 expression.	[101]
miR-3613-3p	BC	Palbociclib	clinical samples; PDTX; cell lines	MiR-3613-3p induce G1 cell cycle arrest and enhance TNBC sensitivity to palbociclib.	[282]
miR-29b-3p	HER-negative BC	Palbociclib	clinical samples; PDTX; cell lines	MiR-29b downregulate CDK6 and induce cell cycle arrest at G1 phase. Activated c-Myc downregulate miR-29b, further activate CDK6 and resistance to palbociclib.	[203]
miR-497	ALCL	Palbociclib	cell lines	Hypermethylation repressed miR-497 expression. miR-497 inhibited CDK6, E2F3 and cyclin E1 (G1) caused cell cycle arrest. higher miR-497 expression cell more sensitive to palbociclib	[205]
miR-193b	PCA	Palbociclib	cell lines	MiR-193b downregulate cyclin D1 by targeting 3′UTR of mRNA. palbociclib has no effect on cells with high miR-193b expression.	[209]
miR-200a	MM	Palbociclib	cell lines	MiR-200 reduces CDK6 expression and reduces melanoma response to palbociclib.	[210]
miR-223	lum BC; HER2-positive BC	Palbociclib	transgenic mouse model; clinical samples; cell lines	Palbociclib restrain E2F1 activity and restore miR-223 expression. miR-223 deficiency induces luminal breast cancer resistance to palbociclib.	[283]
miR-126	lum BC; HER2-positive BC	Ribociclib	cell lines.	MiR-126 targets PI3K/AKT/MTOR pathway, affects cell cycle and mitosis. miR-125 preserving leukemia stem cell quiescence and promoting chemotherapy resistance.	[284]
MIR17HG	GBM, AT/RT	Palbociclib; Ribociclib	cell lines	MIR17HG reduce cyclin D1 expression and sensitizes ATRT cells to palbociclib treatment.	[173]
miR-432-5p	ER-positive BC	palbociclib; Ribociclib	cell lines	Palbociclib resistant cells increased miR-432-5p and CDK6 expression. In palbociclib resistance breast cancers, miR-432-5p is higher expressed.	[285]
miR-9	HER2-positive BC; TNBC	Ribociclib;	cell lines	Suppression of CDK6 and enhance the activity of ribociclib.	[284]
miR-326	HER2-positive BC	Ribociclib	cell lines	Suppression of CDK6 and enhance the activity of ribociclib.	[82]
miR-124a	ALL	Palbociclib; PD-0332991	cell lines	MiR-124a was down-regulated in ALL by hypermethylation of the promoter and histone modifications. MiR-124 negatively regulates CDK6 and pRB. ALL patients with overexpressed miR-124a benefit from CDK6 inhibition.	[149]
miR-9	ALL	Palbociclib	cell lines	Epigenetic downregulation of MIR9 induced upregulation of CDK6.	[174]

Abbreviations: LC, lung cancer; BC, breast cancer; TNBC, triple-negative breast cancer; HCC, hepatocellular carcinoma; GBM, glioblastoma; ALCL, anaplastic large cell lymphoma; PCA, prostate cancer; MM, malignant melanoma; lum BC, luminal breast cancer; AT/RT, atypical teratoid/rhabdoid tumor; ALL, acute lymphoblastic leukemia; PDTX, patient-derived tumor xenograft.

## Data Availability

Not applicable.

## Supplementary Materials

The supporting information can be downloaded at: https://www.mdpi.com/article/10.3390/ijms24108939/s1.
