# Supplementary material for: Regulation of the Cell Cycle by ncRNAs Affects the Efficiency of CDK4/6 Inhibition
Source: Int J Mol Sci. 2023 May 18;24(10):8939. doi: 10.3390/ijms24108939 (PMC10219488; doi:10.3390/ijms24108939)
Supplement: Supplementary file 1 [file ijms-24-08939-s001.zip › ijms-2324469-supplementary.pdf]

Review

Regulation of Cell Cycle by ncRNA Affects the Efficiency of CDK4/6 Inhibitions

Qingyi Hu and Tao Huang*

Table S1. CDK4/6 inhibitions (palbociclib, abemaciclib, and ribociclib) treatment clinical trails in human malignant tumors.

Trail ID	Phase	Enrolled Cancer patients	N	Regimen
NCT03709680	I/II	relapsed or refractory solid tumor or EWS	184	Palbociclib + Temozolomide + Irinotecan v.s. Temozolomide + Irinotecan
SERENA-6 (NCT04964934)	III	HR-positive, HER2-negative MBC with detectable ESR1 mutation	300	AZD9833 + CDK4/6i (Palbociclib or Abemaciclib) v.s. AI (Anastrozole or Letrozole) + CDK4/6i
NCT02738866	II	HR-positive, HER2-negative BC metastatic or locally advanced	60	Palbociclib + Fulvestrant
INCMGA00012 (NCT04438824)	II	Liposarcoma metastatic or unresectable	42	Palbociclib + INCMGA00012
NCT02778685	II	Metastatic Stage IV ER-positive BC	47	Palbociclib + Pembrolizumab + Letrozole
VIKTORIA-1 (NCT05501886)	III	Advanced or Metastatic HR-positive, HER2-negative BC	701	Palbociclib + Gedatolisib + Fulvestrant v.s. Gedatolisib + Fulvestrant
NCT05694871	II	Advanced Dedifferentiated Liposarcoma	72	Palbociclib v.s. Palbociclib + Cemiplimab
IBRANCE (NCT04072952)	I/II	HR-positive, HER2-negative Locally Advanced or MBC	215	ARV-471 v.s. ARV-471 + Palbociclib
NCT04191499	II/III	PIK3CA-Mutant, HR-positive, Her2-negative, Locally Advanced or MBC	400	Inavolisib + Palbociclib + Fulvestrant v.s. Placebo + Palbociclib + Fulvestrant
NCT04711252	III	ER-positive, HER2-negative BC and Not Received Any Systemic Treatment	1342	AZD9833 + Palbociclib v.s. Anastrozole + Palbociclib
PALAVY (NCT04841148)	II	Stage II-III BC	96	Avelumab v.s. Hydroxychloroquine v.s. Avelumab + Palbociclib v.s. Hydroxychloroquine + Palbociclib
NCT03446157	II	wild-type metastatic colorectal cancer	24	Palbociclib + Cetuximab
NCT03573648	II	Stage I-III ER-positive BC	33	Avelumab + ET + palbociclib v.s. Avelumab + ET
NCT05384119	I/II	Metastatic HR-positive, HER2-negative BC	53	TTI-101 + Palbociclib + AI
NCT02896335	II	any solid tumor with Recurrent Brain Metastases	30	Palbociclib
NCT02760030	II	Older Patients with HR-ponsive and inoperable BC	37	Fulvestrant + Palbociclib
NCT03065062	I	Advanced Solid Tumors or Lung, Pancreatic, or Head and Neck Cancer	96	Palbociclib + Gedatolisib
CAPItello-292 (NCT04862663)	III	HR-positive, Her2-negative Locally Advanced, Unresectable or MBC	700	Capivasertib + CDK4/6i + Fulvestrant v.s. Placebo + CDK4/6i + Fulvestrant
NCT03535506	II	Stage 0-I Ductal BC in Situ	24	Palbociclib before Surgery
NCT03170206	I	Advanced KRAS Mutant Non-small Cell Lung Cancer	72	Palbociclib v.s. Palbociclib + Binimetinib v.s. Binimetinib
NCT04966481	III	CDKN2A-altered, HPV-unrelated Recurrent or Metastatic HNSC	81	Palbociclib + Cetuximab v.s. Cetuximab

INTERACT (NCT04256941)	II	HR-positive MBC with an ERS1 Activating Mutation	124	Letrozole, Anastrozole, or Fulvestrant with CDK4/6i (Ribociclib, Palbociclib, Abemaciclib)
NCT05069038	II	Early-stage HR-positive, HER2/neu-negative BC	46	Neoadjuvant Palbociclib + ET (letrozole and goserelin)
MK-6482-024 (NCT05468697)	I/II	Advanced Renal Cell Carcinoma	180	Belzutifan (MK-6482) + Palbociclib v.s. Belzutifan
HPOE (NCT03685331)	I/II	BRCA Mutation-Associated, HR-positive, HER2-negative ABC	54	Olaparib, Palbociclib, and Fulvestrant
NCT03132454	I	Recurrent or Refractory Leukemia	54	Palbociclib, Sorafenib, Decitabine, and Dexamethasone
NCT03454035	I	Advanced or Refractory Solid Tumors or Metastatic Pancreatic Cancer or Metastatic Melanoma	45	Ulixertinib + Palbociclib
RELPALL2 (NCT04996160)	I	Relapsed or Refractory Acute Lymphoblastic Leukemia	22	Palbociclib, Bortezomib, Dexamethasone, and Doxorubicin
NCT03065387	I	Refractory and Advanced or Metastatic Solid Tumors with EGFR Mutation/Amplification, HER2 Mutation/Amplification, or HER3/4 Mutation or KRAS Mutation	120	Neratinib, Everolimus, Palbociclib, or Trametinib
NCT04870034	I	Operable KRAS-positive Lung, Colorectal, or Pancreatic Cancer	24	Binimetinib and Palbociclib before Surgery
NCT04563507	II	ER-positive MBC	102	letrozole+palbociclib + SBRT
Combo-MATCH (NCT05554367)	II	RAS-Mutant Cancers	199	Palbociclib, Binimetinib
NCT05665361	I/II	Advanced ccRCC or pRCC	100	Palbociclib, Sasanlimab
NCT05627232	I	Relapsed or Refractory AML	15	Palbociclib + CPX-351 + Tazemetostat
NCT05176288	II	Advanced, Metastatic, or Unresectable Clear Cell Kidney Cancer	25	Avelumab, Axitinib, and Palbociclib
NCT03774472	I/II	ER-positive, HER2-negative BC	54	Hydroxychloroquine, Palbociclib, and Letrozole before Surgery
NCT02523014	II	Progressive Meningiomas	124	Vismodegib, FAK Inhibitor GSK2256098, Capivasertib, and Abemaciclib
NCT05169567	III	HR-positive, HER2-negative BC that progressed or recurred after previous treatment	350	Abemaciclib + Fulvestrant v.s. Fulvestrant + Placebo
NCT03939897	I/II	HR-positive, HER2-negative stage IV BC	204	Copanlisib + Abemaciclib + Fulvestrant
SARC041 (NCT04967521)	III	Advanced Dedifferentiated Liposarcoma	108	Abemaciclib v.s. Placebo
NCT04751929	II	Metastatic Castration Resistant Prostate Cancer	75	Abemaciclib v.s. Abemaciclib + Atezolizumab
SERENA-6 (NCT04964934)	III	HR-positive, HER2-negative MBC With Detectable ESR1m Before Progression	300	AZD9833 + CDK4/6i (Palbociclib or Abemaciclib) v.s. Anastrozole + CDK4/6i v.s. AZD9833 + Placebo
NCT04633239	I	Recurrent Ovarian Cancer	42	Abemaciclib + Olaparib
NCT03643510	II	HR-positive Recurrent or Refractory Endometrial Cancer	25	Fulvestrant + Abemaciclib
NCT05714072	I	Myelofibrosis	18	Ruxolitinib + Abemaciclib
NCT05288166	III	Metastatic Hormone-Sensitive Prostate Cancer	900	Abemaciclib + Abiraterone
NCT04393285	II	Advanced stage, persistent or recurrent endometrioid endometrial cancer	50	Abemaciclib + Letrozole
NCT03675893	II	Recurrent, Metastatic, or Resistant Endometrial Cancer	60	Abemaciclib, Letrozole, LY3023414, and Metformin

NCT05095207	I/II	Locally Advanced, Unresectable, or Metastatic AR-positive, HER2-negative BC	60	Abemaciclib + Bicalutamide
NCT04975308	III	ER- positive, HER2-negative BC	60	Abemaciclib + Bicalutamide
NCT04040205	II	Bone and Soft Tissue Sarcoma	45	Abemaciclib
NCT03979508	II	Surgically Resectable, Chemotherapy Resistant, TNBC	200	Abemaciclib v.s. Pembrolizumab + Abemaciclib
NCT04627064	I	Advanced Kidney Cancer	40	Abemaciclib v.s. Abemaciclib + MK-6482
NCT02644460	I	Diffuse Intrinsic Pontine Glioma, Recurrent or Refractory Solid Tumors, or Malignant Brain Tumors	60	Abemaciclib + Radiation
NCT05065411	III	ER-positive, HER2-negative MBC	186	Abemaciclib + Enobosarm
AIM (NCT04169074)	II	HPV-negative Stage I-IVA HNSC Undergoing Surgery	20	Abemaciclib
NCT03310879	II	Advanced Solid Tumors with CCND1/2/3 and CDK4/6 Genetic Alterations	38	Abemaciclib
INSIGhT (NCT02977780)	II	Brain Tumors after Biomarker Screening	250	Abemaciclib, DNA-PK/TOR Kinase Inhibitor CC-115, Neratinib, or QBS10072S
NCT04791384	I	Brain Metastasis Due to HR-positive, HER2-negative BC	44	Abemaciclib + Elacestrant
NCT05608252	I/II	Metastatic or Locally Recurrent HR-positive, HER2-negative BC	63	Abemaciclib, Avutometinib, and Fulvestrant
NCT04049227	I	Endometrial Cancer	27	Abemaciclib + Letrozole
NCT04010357	II	Rb Wild-Type Refractory or Relapsed Extensive Stage SCLC	29	Abemaciclib
NCT03891784	II	Advanced, Refractory, and Unresectable Digestive System Neuroendocrine Tumors	37	Abemaciclib
ABBY (NCT04523857)	II	BC	66	Abemaciclib v.s. Abemaciclib + Hydroxychloroquine
NCT04534283	II	Advanced, Metastatic, or Unresectable Cancer Harboring Mutations in BRAF, RAF1, MEK1/2, ERK1/2, and NF1	35	ERK1/2 Inhibitor LY3214996 + Abemaciclib
NCT04545710	II	Stage IV or Recurrent NSCLC with EGFR Activating Mutations with Osimertinib Resistance	18	Osimertinib + Abemaciclib
NCT04469764	II	Recurrent Ovarian or Endometrial Cancer	32	Abemaciclib
RAD 1805 (NCT04298983)	II	Localized High-Risk or Locally Advanced Prostate Cancer	30	Abemaciclib + ADT
NCT04614194	II	HR-positive BC	60	Neoadjuvant Letrozole + Abemaciclib v.s. Letrozole
NCT05524584	II	HR-positive, HER2-negative MBC	20	Anastrozole, Fulvestrant, and Abemaciclib
NCT04750928	I/II	NF1 Related Atypical Neurofibromas	50	Abemaciclib
NCT05113537	I/II	Metastatic Castrate Resistant Prostate Cancer	30	Abemaciclib before 177Lu-PSMA-617
NCT05386108	I/II	Brain Metastasis Due to HR-positive, HER2-negative BC	106	Abemaciclib + Elacestrant
NCT04923542	I/II	HR-positive, HER2-negative MBC	31	Stereotactic Radiosurgery, Abemaciclib, and ET
NCT04941274	I/II	HIV-Associated and HIV-negative Kaposi Sarcoma	43	Abemaciclib
NCT04238819	I/II	Children and Young Adult Participants with Solid Tumors, Including Neuroblastoma	117	Abemaciclib, Irinotecan, Temozolomide, Dinutuximab, and GM-CSF
NCT04616183	I/II	Unresectable or Metastatic Colorectal Cancer	46	LY3214996 + Cetuximab v.s. LY3214996 + Cetuximab + Abemaciclib
NCT05413304	I	Recurrent High-Grade Glioma	7	Abemaciclib

NCT04352777	IV	Locally Advanced, Unresectable or Metastatic ER-positive, HER2-negative BC	18	ET and Abemaciclib
NCT05372640	I	Adult and Pediatric Patients (12-17 years) with Metastatic or Unresectable NUT Carcinoma and Other Solid Tumors	30	ZEN003694 + Abemaciclib
NCT04552769	II	Unresectable or Metastatic Anaplastic/Undifferentiated Thyroid Cancer	9	Abemaciclib
NCT03285412	II	ER-positive BC	120	Ribociclib
NCT03008408	II	Advanced or Recurrent Endometrial Carcinoma	87	Everolimus + Letrozole + Ribociclib v.s. Everolimus + Letrozole
LEANORA (NCT04657679)	IV	ABC in Race-Based Cohorts	36	Ribociclib
NCT05467891	II	Locoregional Recurrent, Resected HR-positive, HER2-negative BC	200	Ribociclib, ET
NCT03090165	I/II	Advanced AR-positive TNBC	37	Ribociclib + Bicalutamide
Charge (NCT04315233)	I	Metastatic TNBC or Recurrent Platinum-Resistant Ovarian Cancer	34	Ribociclib + Belinostat
NCT05508906	I	ABC or MBC	60	Ribociclib + Alpelisib
NCT03056833	I	Recurrent Ovarian, Fallopian Tube, or Primary Peritoneal Cancer	35	Ribociclib + Carboplatin + Paclitaxel
NCT05319873	I/II	HER2-positive BC	18	Ribociclib, Tucatinib, and Trastuzumab
NCT03740334	I	Children and Young Adults with Relapsed or Refractory Acute Lymphoblastic Leukemia	45	Ribociclib + Everolimus + Dexamethasone
MONARCH 2 (NCT02107703)	III	HR-positive, HER2-negative ABC	669	Abemaciclib + Fulvestrant
MONARCH 3 (NCT02246621)	III	HR-positive, HER2-negative ABC previously treated with ET	493	Abemaciclib v.s. Abemaciclib + Fulvestrant

Abbreviations: N, actual or estimated enrollment number; EWS, Ewing sarcoma; HR, hormone receptor; HER2, human epidermal growth factor receptor 2; MBC, metastatic breast cancer; BC, breast cancer; ER, estrogen receptor; HNSC, Head and Neck Squamous Cell Carcinoma; ccRCC, Clear Cell Renal Cell Carcinoma; pRCC, Papillary Renal Cell Carcinoma; AML, Acute Myeloid Leukemia; AR, Androgen Receptor; TNBC, Triple Negative Breast Cancer; SCLC, Small Cell Lung Cancer; NSCLC, Non-small Cell Lung Cancer; NF1, Neurofibromatosis Type I; ET, Endocrine therapy; AI, aromatase inhibitor; CDK4/6i, CDK4/6 inhibitor; SBRT, Stereotactic Body Radiation Therapy; ADT, Androgen Deprivation Therapy; ABC, advanced breast cancer.
